# Electrical stimulation through conductive scaffolds for cardiomyocyte tissue engineering: Systematic review and narrative synthesis

**DOI:** 10.1111/nyas.14812

**Published:** 2022-06-08

**Authors:** Louie Scott, Katrín Elídóttir, Kamalan Jeevaratnam, Izabela Jurewicz, Rebecca Lewis

**Affiliations:** ^1^ School of Veterinary Medicine University of Surrey Guildford UK; ^2^ Department of Physics University of Surrey Guildford UK

**Keywords:** biomaterials, cardiac tissue engineering, direct coupling, electrical stimulation, systematic review

## Abstract

Electrical conductivity is of great significance to cardiac tissue engineering and permits the use of electrical stimulation in mimicking cardiac pacing. The development of biomaterials for tissue engineering can incorporate physical properties that are uncommon to standard cell culture and can facilitate improved cardiomyocyte function. In this review, the PICOT question asks, “How has the application of external electrical stimulation in conductive scaffolds for tissue engineering affected cardiomyocyte behavior in *in vitro* cell culture?” The Preferred Reporting Items for Systematic Reviews and Meta‐Analysis guidelines, with predetermined inclusion and quality appraisal criteria, were used to assess publications from PubMed, Web of Science, and Scopus. Results revealed carbon nanotubes to be the most common conductive agent in biomaterials and rodent‐sourced cell types as the most common cardiomyocytes used. To assess cardiomyocytes, immunofluorescence was used most often, utilizing proteins, such as connexin 43, cardiac α‐actinin, and cardiac troponins. It was determined that the modal average stimulation protocol comprised 1–3 V square biphasic 50‐ms pulses at 1 Hz, applied toward the end of cell culture. The addition of electrical stimulation to *in vitro* culture has exemplified it as a powerful tool for cardiac tissue engineering and brings researchers closer to creating optimal artificial cardiac tissue constructs.

## Graphical abstract

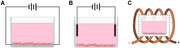

## INTRODUCTION

It can be said that the two goals of tissue engineering (TE) are: to create engineered tissues that can be transplanted into the body to restore function to damaged or diseased organ systems, as a tissue construct or as a vehicle for functional cells; and to create biomimetic platforms that enable accurate and reliable study of cells and tissues *in vitro*.^1^, ^2^, ^3^, ^4^ To achieve these objectives, TE aims to closely mimic the stimuli that cells experience *in vivo*—chemical, mechanical, physical, or otherwise. Cardiovascular disease (CVD) research is one area which could greatly benefit from the implementation of more TE methodologies.^5^ According to the WHO, approximately 17.9 million people die annually as a result of CVDs, which translates to 31% of all deaths globally.^6^ It is also predicted that by 2030, the annual number of deaths as the result of CVDs will reach 24.2 million.^7^ Despite CVD's continued burden on healthcare services, between 1990 and 2012, there was a general decline in the amount of research into CVDs.^8^ While research into CVDs is starting to increase again, basic research—interrogating the mechanisms behind CVDs—has shown the least growth.^9^ Part of this stagnation in research can be attributed to the challenge of culturing cardiac cell types *ex vivo*, resulting in less effective and reliable testing of new treatments, which, in turn, can lead to a high rate of late‐stage attrition when attempting to get novel therapies approved.^10^

The cardiovascular system is highly specialized to perform its function and relies on continuous maintenance of nutritional, electrical, and mechanical stimuli to continue to do so.^11^ When this homeostasis is interrupted, in ischemic events, like myocardial infarction, where blood flow to a section of the heart is blocked, a steep and naturally unrecoverable decline is observed in cell function, with many cells dying and being replaced by scar tissue in the form of cardiac fibroblasts.^12^, ^13^ Similarly, if stimuli equivalent to those observed *in vivo* are not provided *in vitro*, then unsuccessful attempts to culture healthy, functional cardiac tissues can be expected.^10^ Traditionally, cell culture for adherent cell types consists of seeding cells on a flat, plasma‐treated or protein‐coated surface, providing nutrients in the form of liquid media, and maintaining temperature at 37°C and atmospheric composition of CO₂ at 5%.^14^, ^15^, ^16^ In this setup, key features of the cardiac cellular microenvironment are missing, such as physical or chemical cues to guide growth, matching mechanical properties of the myocardium, and, perhaps most importantly, frequent and rhythmic electrical stimulation.^17^, ^18^ TE is uniquely equipped to provide solutions to these missing stimuli and bring about the creation of novel and dynamic cell culture platforms to satisfy the exacting requirements of cardiac cell types.

**FIGURE 1:**
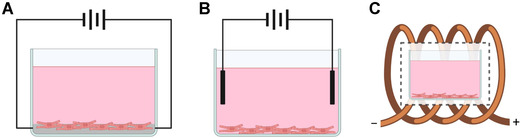
In vitro electrical stimulation methodologies. Illustrations of the three methods by which electrical stimulation can be delivered *in vitro*: (A) direct coupling, (B) capacitive coupling, and (C) inductive coupling. This figure was created with BioRender.com and adapted from Ref. 26

When considering electrical stimulation's role in *in vitro* cell culture and TE, it is important to acknowledge the electrical properties of cells and their contents. In this context, and in terms of electrophysiology, cells derive much of their electrical properties from their membrane and, in turn, the membrane derives its properties from the lipids and proteins of which it is composed.^19^ In cardiac muscle, as with other tissues, protein structures like ion channels and transporters maintain and alter the electrical potential that exists between the exterior and interior of the cell, which at rest is approximately −90 mV in cardiomyocytes (CMs).^19^, ^20^ Initiated by electrogenic pacemaker cells in the sinoatrial node, action potentials flow through the cardiac muscle, propagated from one cell to the next by intercalated disc protein structures known as gap junctions, which permit the passage of ions between adjacent cells, thus allowing for rhythmic and synchronous contractions throughout the tissue.^21^, ^22^, ^23^, ^24^ Cardiac action potentials are what initiate contractions in CMs and can simply be described as a rapid depolarization and controlled repolarization of the membrane potential by active transport of sodium, calcium, and potassium ions across the membrane.^25^ Cardiac action potentials occur between 60 and 80 times per minute in humans and act as a constant source of stimulus for CMs. As such, this stimulation has a key role in the growth, development, maintenance, and even dysfunction of cardiac muscle—which is why the absence of electrical stimulation in the majority of cardiac TE cannot be overstated. When electrical stimulation is present in cell culture, it can be applied in three different ways (Figure 1). In direct coupling, electrodes are attached to the tissue or scaffold to deliver electrical stimulation, and while this method is perhaps the most efficient delivery method and most representative of *in situ*, stimulating electrodes and conductive materials frequently suffer from poor biocompatibility.^26^ Capacitive coupling uses two electrodes at opposite ends and the culture media as the conductor to provide an even electric field across the culture chamber.^26^ While this method is biologically safer, the inefficiency of liquid media as conductor can require higher voltages, which can lead to increases in temperature and production of toxicants at the electrodes.^27^ Finally, inductive coupling uses a conductive coil to encapsulate the cell culture chamber to create a pulsed electromagnetic field, though this method is fairly uncommon due to the specialist equipment and expertise required and high consumption of resources.^26^

Direct coupling stimulation shows the most potential for delivering representative electrical stimulus to *in vitro* engineered tissues in cardiac TE. The growing field of cardiac TE also shows great promise in providing solutions to the logistical, design, and biological issues with this form of electrical stimulation for cardiac muscle tissue constructs. With many different researchers working independently of one another, using different materials, designs, and conditions, it can be difficult to decipher the true effects of electrical stimulation and how it could be incorporated into regular cardiac cell culture practices. Hence, this review aims to characterize and summarize the published findings of research that has chosen to employ conductive scaffolds and direct coupling stimulation for CM cell culture. Through a systematic approach, we will assess the effectiveness of conductive biomaterials in CM culture, the optimal protocol parameters for electrical stimulation, where and how these materials and techniques can be best applied in cardiac TE, and, finally, future directions of these practices and their place in TE research.

## REVIEW METHODOLOGY

### Objectives

This systematic review aims to characterize the effects of external electrical stimulation through conductive biomaterial scaffolds to identify experimental parameters that most affect cell function and discuss how the use of this technique could advance current research.

### Methods

The research question summarizing this review was developed using the PICOT format^28^ and is as follows: How has the application of external electrical stimulation in conductive scaffolds for TE affected CM behavior in *in vitro* cell culture? The Preferred Reporting Items for Systematic Reviews and Meta‐Analysis guidelines were then followed to conduct the systematic review and narrative synthesis.^29^

### Search strategy

In March 2021, PubMed, Web of Science, and Scopus were used to search for publications using the search terms “cardiomyocyte,” “cardiac myocyte,” “electrical stimulation,” and “scaffold.” Search terms were developed to isolate relevant publications and terms were combined into a phrase in order to focus searches further. The phrase developed is as follows: (“cardiomyocyte*” OR “cardiac myocyte*”) AND “electrical stimulation” AND “scaffold*,” with the format of the phrase being maintained between databases. Where possible, the searches were applied to all fields and databases, ensuring as many areas in each database were searched as possible.

### Search attrition criteria

The results of each phrase from each database were first sorted based on exclusion criteria. The criteria were applied as follows: the paper must be a primary research publication—no reviews, editorials, books, patents, or conference reports; the paper, or a version of, must be published in English; and the paper must be open access.

The resulting papers were then screened against the inclusion criteria, which focused on the topic of the paper based on the title and abstract. The criteria center around the research question: How has the application of electrical stimulation in conductive biomaterial scaffolds positively affected CM culture outcomes in TE? This was distilled into the following criteria: must include the use of external electrical stimulation; must include the use of conductive tissue culture scaffolds; and must include the use of CMs—no other cardiac cell types or stem cells.

### Article processing and selection

Once the exclusion and inclusion criteria had been applied to all the search results and duplicates were removed, the final publication library was reviewed by two investigators. The full texts were obtained from the respective publishers. If the full publications were not available through open access, the authors were contacted via the paper's correspondence email. Failing that, the paper was excluded from the final review set.

### Quality appraisal

As laid out by the Task Force of Academic Medicine and GEA‐RIME committee, the Checklist of Review Criteria was used on the final review library to assess the quality and relevance of the papers as a whole, as opposed to assessing the title and abstract alone.^30^ By using this framework, each section of the publications can be assessed for scientific merit as well as features that should run through the entirety of the paper, such as well‐identified research problems, robust experimental design, and critical data analysis. The Checklist of Review Criteria categories are as follows and numbers correspond to the columns in Table 1:

1. Problem statement, conceptual framework, and research question
2. Reference to the literature and documentation
3. Relevance
4. Research design
5. Instrumentation, data collection, and quality control
6. Population and sample
7. Data analysis and statistics
8. Reporting of statistical analyses
9. Presentation of results
10. Discussion and conclusion: interpretation
11. Title, authors, and abstract
12. Presentation and documentation
13. Scientific conduct

**TABLE 1:** Quality appraisal of the 12 publications retrieved after database inception and search attrition. (1) Problem statement, conceptual framework, and research question, (2) Reference to literature and documentation, (3) Relevance, (4) Research design, (5) Instrumentation, data collection, and quality control, (6) Population and sample, (7) Data analysis and statistics, (8) Reporting of statistical analyses, (9) Presentation of results, (10) Discussion and conclusion: interpretation, (11) Title, authors, and abstract, (12) Presentation and documentation, (13) Scientific conduct

	Categories of Checklist of Review Criteria													
Publication	1	2	3	4	5	6	7	8	9	10	11	12	13	Total criteria met
Allison^50^	✓	✓	✓	✓	✓	✓	✓	✓	✓	✓	✓	✓	✓	13
Feiner^36^	✓	✓	✓	✓	✓	✓	✓	✓	✓	✓	✓	✓	✓	13
Ganji^79^	✓	✓	✓	✓	✓	✓	✓	✓	✓		✓	✓	✓	12
Hsiao^80^	✓	✓	✓	✓	✓	✓	✓	✓	✓		✓	✓	✓	12
Kai^34^	✓	✓	✓	✓	✓	✓	✓	✓	✓	✓	✓	✓	✓	13
Navaei^33^	✓	✓	✓	✓	✓	✓	✓	✓	✓		✓	✓	✓	12
Ren^31^	✓	✓	✓	✓	✓	✓	✓	✓	✓	✓	✓	✓	✓	13
Shin^37^	✓	✓	✓	✓	✓	✓	✓	✓	✓		✓	✓	✓	12
Shin^40^	✓	✓	✓	✓	✓	✓	✓	✓	✓		✓	✓	✓	12
Shin^38^	✓	✓	✓	✓	✓	✓	✓	✓	✓	✓	✓	✓	✓	13
Wang^35^	✓	✓	✓	✓	✓	✓	✓	✓	✓	✓	✓	✓	✓	13
You^39^	✓	✓	✓	✓	✓	✓	✓	✓	✓		✓	✓	✓	12
Total papers														12

### Data extraction

For this review, a specialized data extraction table was designed to collate and summarize the key information from each paper. The main categories in the table included: biological materials used (type of cells used and their source), synthetic materials used (additional scaffold biomaterials and scaffold fabrication method), electrical stimulation protocol, techniques used to assess CM viability and function, key results and discussion, limitations identified by authors, and future work suggested by authors.

## RESULTS

### Search breakdown

**FIGURE 2:**
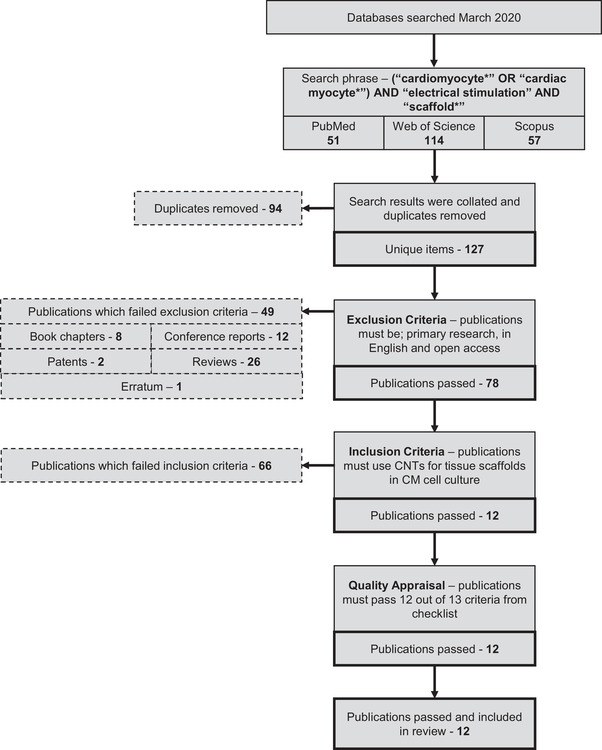
Search attrition. Publication attrition process depicted in a step‐by‐step flowchart that details the method by which the final set of reviewed publications was produced. PubMed, Web of Science, and Scopus publication were searched using terms relating to electrical stimulation in conductive scaffolds for cardiomyocyte cell culture. After removing duplicate items, exclusion and inclusion criteria were applied to isolate primary research publications specifically relating to the review question

**FIGURE 3:**
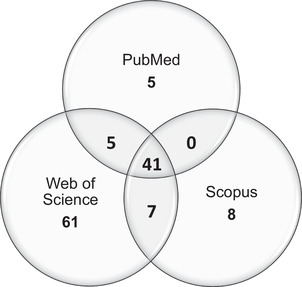
Database overlap. Venn diagram depicting the sources of unique publications from database searches, and the overlap between the different chosen publication databases: PubMed, Web of Science, and Scopus

The literature search generated 51 publications from PubMed, 114 from Web of Science, and 57 from Scopus (Figures 2 and 3). Forty‐two percent of the total 222 papers were duplicates and were subsequently removed. From the remaining 127 papers, 49 were removed based on the exclusion criteria and 66 were removed based on the inclusion criteria, leaving 12 publications, which were acceptable for quality appraisal.

### Quality appraisal

The 12 publications that remained after search attrition were assessed using 13 criteria. Papers which did not pass at least 12 of the 13 criteria were rejected from further consideration (Table 1). All publications passed the quality appraisal process and were deemed fit for inclusion in this systematic review. The contents of all 12 publications were then summarized (Table 2).

**TABLE 2:** Summarized contents of all 12 reviewed publications

	Materials used							
	Biological		Synthetic					
Publication	Cells	Source	Scaffold material and design	Electrical stimulation protocol	Techniques used to assess CM viability and function	Results and discussion	Limitations suggested by the authors	Further work suggested by the authors
Allison^50^	Neonatal ventricular CMs	2‐day‐old Sprague–Dawley rat pups	Silver nanoparticle (AgNP)‐doped, electrospun, aligned collagen fibers	1‐V, 5‐ms pulse, 5 Hz for 24 h via C‐PACE system	Confocal IF for α‐actinin, Cx43, and Ki67 plus DAPI; flow cytometry for Cx43	No change in α‐actinin expression between scaffolds with and without AgNPs. Cx43 expression doubled in paced, AgNP scaffolds, confirmed by IF and FC. Ki67 expression increased with AgNPs, more so with ES.	Mechanism behind Ki67 increase is unclear	*In vivo* testing and assessment of nanosilver size and shape on scaffold conductivity
Feiner^36^	Neonatal left ventricular CMs	0‐ to 3‐day‐old Sprague–Dawley rat pups	Gold‐ and TiN‐coated SU‐8/AZ 5214 photoresist polymer matrix patch	3‐V, 50‐ms pulse, 1 and 2 Hz	Confocal IF of α‐actinin plus DAPI; PrestoBlue viability assay; ePhys recording via scaffold; pStim with adrenaline; calcium imaging	Increased elongation, aspect ratio, and striation from α‐actinin IF. Scaffold components had no effect on viability. pStim caused two‐fold increase in action potential frequency. CM conduction velocity similar to other engineered CM tissues *in vitro*. ES activated noncontractile cells, increased synchronicity, and allowed the manipulation of rhythm and direction of contractions.	The scaffolds alone fail to create fully contractile and synchronous cultures	Creating stretchable scaffolds to improve mechanical stability and adding pH, heat, and mechanical stress‐sensing capabilities
Ganji^79^	H9C2 CMs	Clonal cell line derived from embryonic rat heart tissue	Polyurethane‐gold nanotubes/nanowires (GNT/NW) composite	1 V/mm, 2‐ms pulse, 1 Hz for 15 min for 3 days	Calcein AM/Hoechst 32258 cell viability staining; qRT‐PCR after ES for ANF, GAPDH, β‐MHC, NPPB, GATA4, B2M, TBP, 18 sr RNA, Cx43, cTnI, TnT2, Nkx2.5, and Mef2c; SEM	Increased adhesion and growth on gold‐containing scaffolds. Increased alignment and confluency, with further increase after ES. Housekeeping genes GAPDH, B2M, TBP, and 18 sr RNA showed no increase, with or without ES. Scaffolds with 50 ppm GNT/NW showed greatest increase in all other transcription factors. SEM confirmed results from staining.	No limitations suggested	No future research suggested
Hsiao^80^	Neonatal cardiomyocytes	1‐ to 2‐day old Lewis rat pups	HCl‐doped nanofibrous mesh of PANI/PLGA electrospun fibers	Trains of pulses at 5 V/cm and 1.25 Hz	Live/Dead assay; LDH leakage assay; IF of cTnT and Cx43 plus PI; beating analysis from fluorescent imaging of DiI‐labeled cells	Doped scaffolds showed similar viability to controls, but undoped displayed decrease due to poor cell adherence. Increased elongation and alignment in doped scaffolds. High expression of cTnT and Cx43 in clusters of CMs. ES promoted rhythmic and synchronous contractions in and between clusters.	Full cell confluence was not achieved, CMs grew in individual clusters	No future research suggested
Kai^34^	Adult ventricular cardiomyocytes	Primary cells isolated from adult human heart	Nanofibrous mesh of melanin/PLCL/gelatin electrospun fibers	Trains of rectangular 150‐ms pulses at 1 V/cm and 1 Hz for 1 h for 3 days	MTS proliferation assay; Live/Dead assay; phalloidin F‐actin staining plus DAPI; confocal IF of α‐actinin and Cx43; repeat MTS, F‐actin staining, and Cx43 IF after ES	Cell proliferation and viability decreased as [melanin] increased. Increased adherence, growth, and alignment on low melanin scaffolds. High expression of α‐actinin on low melanin scaffolds and highest expression of Cx43 on 10% melanin scaffold. After ES, increased proliferation, Cx43 expression, and CMs elongated and aligned in the direction of the ES field.	Addition of melanins weakened the mechanical properties of the scaffolds. Melanins exhibit dose‐dependent cytotoxicity.	Alternative fiber production, like coaxial, and using more conductive melanins
Navaei^33^	Neonatal ventricular cardiomyocytes	2‐day‐old rat pups	GelMA‐gold nanorod (GNR) gelatin hydrogel discs	V @ excitation threshold, 2‐ms pulse, 1, 2, or 3 Hz	Live/Dead assay; Alamar Blue assay; phalloidin F‐actin staining plus DAPI with FFT analysis of alignment; confocal IF of α‐actinin and Cx43; IF of cTnI and integrin β1, custom beating analysis software, Fluo‐4 AM calcium imaging	Increased cell retention, adherence, and viability on GNR scaffolds. No change in metabolic activity between scaffolds. Increased alignment and expression of F‐actin with high [GNR]. Increased expression and organization of α‐actinin, cTnI, Cx43, and integrin β1. Increased spontaneous and synchronous contractions with higher BPM. Lower excitation threshold on high [GNR] scaffolds.	No limitations suggested	Applying scaffolds to *in vivo* studies
Ren^31^	Neonatal cardiomyocytes	1‐ to 3‐day‐old Sprague–Dawley rat pups	Superaligned carbon nanotube sheets on glass or PDMS	2 V/cm, rectangular 2‐ms pulse, 1 Hz via pacemaker	Apoptosis TUNEL assay; confocal IF of α‐actinin + DAPI; IF of Cx43; contractile analysis by ImagePro Plus; whole‐cell patch clamp	TUNEL assay showed decreased cell death on SA‐CNTs. CMs on SA‐CNTs showed increased alignment and elongation by α‐actinin IF and DAPI. eStim by pacemaker showed no changes in cell morphology or viability. Increased expression of Cx43 and improved localization to gap junctions was shown on SA‐CNTs. Increased spontaneous and synchronized beating was seen on SA‐CNTs. Patch clamp showed a decrease in resting membrane potential and action potential duration with an increase in AP amplitude. Variability in AP duration was also reduced between cells and consecutive beats on SA‐CNTs.	Short CNTs can penetrate the cell membrane and disrupt cell activity	Investigate biodegradable alternatives to PDMS and the application of scaffolds in cardiac resynchronization therapy
Shin^37^	Neonatal ventricular cardiomyocytes	2‐day‐old Sprague–Dawley rat pups	GelMA UV‐cured hydrogel thin films made with 0, 1, 3, and 5 mg/ml multi‐walled CNT concentrations	0–7 V/cm, biphasic square 50‐ms pulse; 0.5, 1, 2, and 3 Hz	Live/Dead assay; MTS/qDNA assay; F‐actin + DAPI stain; confocal IF of α‐actinin, troponin I, and Cx43; WB for α‐actinin, troponin I, and Cx43; SEM; custom software beating analysis; pStim by 1‐heptanol and doxorubin	CNT scaffolds showed increased cell retention and viability by Live/Dead assay. MTS and DNA quantification showed limited proliferation, indicating that CMs are growing but fibroblasts are not dividing. Staining of F‐actin showed improved alignment of whole cells and internal protein organization. Confocal IF showed increased expression of TnI and increased visibility and organization of sarcomeric structures. WB showed an increase in α‐actinin and TnI but only a small increase in Cx43, but IF indicates that Cx43 is instead better localized to gap junctions. CMs also showed increased BPM and beat synchronization. CMs on CNTs showed a reduced threshold potential when undergoing ES. CNT scaffolds exhibited a protective effect against pStim. SEM revealed that CNTs encourage cell elongation and spreading of filopodia.	No limitations suggested	Investigation into pStim resistance on high concentration CNT scaffolds
Shin^40^	Neonatal ventricular cardiomyocytes	2‐day‐old Sprague–Dawley rat pups	Multi‐walled CNT forest electrodes were sandwiched between layers of PEG and multi‐walled CNT‐GelMA hydrogels	1.2 V/cm, square 50‐ms pulse, 0.5–3 Hz	IF of α‐actinin and Cx43 and beating observation	IF showed an increase in sarcomeric α‐actinin organization and alignment but a homogeneous distribution of Cx43 that showed variability with changes in [CNT] and stiffness. CMs showed increased BPM on scaffolds but ES enabled control over synchronous beating.	No limitations suggested	No future research suggested
Shin^38^	Neonatal ventricular cardiomyocytes	2‐day‐old Sprague–Dawley rat pups	Gold electrode sandwiched between micropatterned PEG and GelMA‐multi‐walled CNT hydrogel layers	0.5–6 V, 50‐ms square pulses, 0.5–2 Hz, 2.5–10% duty cycle	Alamar Blue assay; confocal IF of α‐actinin and Cx43 plus DAPI; phalloidin F‐actin staining plus DAPI; custom video beating analysis	CMs adhered and grew in layer throughout the scaffold's microscale grip pattern, forming a pseudo‐3D tissue construct, with high expression and organization of α‐actinin and Cx43. Continued spontaneous and synchronous beating, which caused detachment of the soft robot from the base substrate allowing self‐actuating movement in media. Before addition of the gold electrode, ES was able to control soft robot actuation. With addition of the gold electrode, CMs continued to exhibit high expression α‐actinin and Cx43 with well‐developed F‐actin cross‐striation. Integrated electrodes more efficiently stimulated the scaffolds than “bulk” ES through media and elicit better soft robot movement.	Local stimulation through carbon rod electrodes required higher excitation voltage thresholds. Stimulation at frequencies higher than 1 Hz caused lag in contractions as cells were not able to recover fast enough. Soft robot could not yet generate forward propulsion.	Potential application in regenerative medicine as cardiac or muscle patches
Wang^35^	Neonatal cardiomyocytes	2‐day‐old Sprague–Dawley rat pups	Gold electrode sandwiched between micropatterned PEGDA and GelMA‐multi‐walled CNT hydrogel layers	0.5–6 V, 50‐ms square pulses, 0.5–2 Hz, 2.5–10% duty cycle	Video analysis of cell contractions/soft robot actuation; phalloidin F‐actin staining plus DAPI; confocal IF of α‐actinin and Cx43 plus DAPI	ES provides control over soft robot actuation without harming cardiac tissue. Increased spontaneous beating rates and lower excitation threshold compared to pristine GelMA. Increased elongation and sarcomeric alignment. Higher organization of sarcomeric elements above electrodes.	Some parts of the fabrication process are technically challenging	Optimize fabrication process to improve the ease of production
You^39^	Neonatal cardiomyocytes	Rat pup hearts	Fibronectin‐coated gold nanoparticle‐doped porous thiol‐HEMA/HEMA hydrogel	5 V/cm, 2‐mA, 2‐ms rectangular pulses, 1 Hz	Hoechst 33342 nuclear staining; Alamar Blue assay; confocal IF of Cx43 plus DAPI; SEM; WB of Cx43 and GAPDH	No change in viability but after ES slight increase, though not significant. CMs clustered in microscale pores of non‐ES and ES scaffolds. Beating observed on all scaffolds 5 days after seeding. Increased Cx43 expression after ES, especially in conductive scaffolds, but even without ES, conductive scaffolds showed increase.	Developing tissue repair or replacement strategies depends on greater understanding of cell–matrix and cell–electrical interactions	No future research suggested

Abbreviations: CM, cardiomyocyte; ES, electrical stimulation; ePhys, electrophysiology; IF, immunofluorescence; pStim, pharmaceutical stimulation; WB, western blotting; [X], (concentration).

### Experimental design

Researchers used a wide variety of different methodologies and techniques to create their tissue scaffolds and to assess the state of CMs grown on those scaffolds.

### Experimental materials

**FIGURE 4:**
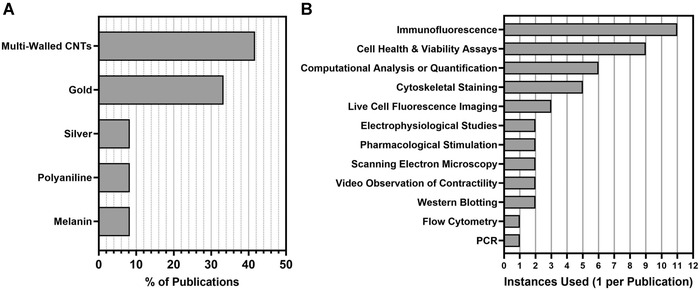
Experimental materials and techniques used. (A) Percentage breakdown of the five types of materials used to achieve conductivity in tissue scaffolds in the reviewed publications. (B) Instances of experimental techniques used in the reviewed publications that were employed to interrogate the health and function of CMs, their interaction with conductive scaffolds, or their reactions to electrical stimulation protocols. Techniques using similar principles and materials or that produced similar data were put into groups

Ninety‐two percent of researchers used rodent CMs, with 91% of those using CMs from neonatal rat pups and of these 60% specified that the CMs were ventricular. One publication used embryonic CMs isolated from stage E18 rat hearts, while another used the H9C2 cell line derived from embryonic rat heart tissue. Only one research group opted for the use of human CMs that were derived from adult ventricular heart tissue. The most common conductive material used in tissue scaffolds, at 42%, was multi‐walled carbon nanotubes (CNTs), and the second most common material was gold at 33% (Figure 4A). Other materials used included silver nanoparticles, polyaniline, and melanin. Additional materials were employed to enable the fabrication of more complex structures and to improve the biocompatibility of conductive reagents. Researchers either used synthetic polymers (33%), hydrogels (33%), or a combination of the two (33%) to form conductive composites.

### Experimental platforms

As previously mentioned, inventive application of biomaterials is one of the things that sets TE apart from traditional *in vitro* cell culture methodologies. Especially when it comes to CMs, scaffold architecture is important to ensure healthy, rhythmically contractile cells and interconnected, synchronous tissue constructs. With regard to this review, it is also important to observe how researchers chose to design their scaffolds to provide optimal delivery of electrical stimulation. Eleven out of the 12 papers identified in this review chose to create homogeneous conductive composites, with 27% of those using electrospinning to create aligned fiber scaffolds. One research group achieved alignment using just pristine multi‐walled CNTs layered on top of poly(dimethylsiloxane) (PDMS), which was used as an inert, nonconductive base.^31^ An additional 27% of researchers using conductive composites cast their scaffolds as flat, solid surfaces with the intention of growing unaligned monolayers of CMs. Another 18% of papers incorporated three‐dimensional design by creating porous scaffolds in which CMs would occupy or line the inside of the pores, perhaps with the intention of mimicking the 3D growth pattern of cells *in vivo*. The remaining three publications used much more complex and intentional designs in their scaffolds to either create soft robots, able to utilize the contractions of CMs to induce repeating actuations of the whole scaffold, or multifunctional patches, capable of monitoring cell cultures and stimulating CMs electrically and pharmacologically.

### Experimental techniques

Numerous techniques were employed by researchers to assess the health and function of CMs on the novel tissue scaffolds, both before and after the application of electrical stimulation protocols (Figure 4B). Immunofluorescence (IF) was the most common technique, being employed by 92% of publications. Of the publications that used IF, connexin 43 (Cx43) was chosen for staining by 91%, while 82% also stained for cardiac α‐actinin. Researchers also used IF to observe the expression of cardiac troponins—key components of the CM contractile machinery—with 18% stained for troponin I and another 9% stained for troponin T. Other proteins stained for include integrin β1 and Ki67. The second most common set of techniques used was health and viability assays, which included, in order of most frequently used: Live/Dead, AlamarBlue, MTS/qDNA, PrestoBlue, LDH leakage, and TUNEL. Other frequently used techniques included computational analysis or quantification using specialist software like ImageJ^32^ or ImagePro Plus (Media Cybernectics, Rockville, MD, USA); cytoskeletal staining, mainly employing phalloidin‐dye conjugates; and live‐cell imaging using reagents, such as calcium‐sensitive dyes.

### Electrical stimulation protocols

As dictated by the review question, publications had to utilize electrical stimulation protocols and specifically by direct coupling through conductive tissue scaffolds. Stimulation protocols varied widely between each publication, though all researchers were aiming to replicate the stimulus CMs would receive *in situ* and improve CM characteristics that indicate cell maturity, contractile functionality, and cell‐to‐cell signaling. Key parameters that describe the protocols include voltage, frequency, pulse duration and shape, and when and for how long stimulation was applied. A third of papers quote the electric field strength used in volts, while the majority, 58%, quoted it as voltage normalized to centimeters or millimeters, with a single paper describing the voltage used as “the minimum required voltage to observe contraction.”^33^ Researchers used a range of frequencies, with many using more than one in different iterations of protocols. Pulse frequency ranged from 0.5 to 5 Hz, with 58% of papers using 1 Hz at some point. Forty‐two percent of protocols used 50‐ms pulses, while another 33% used only 2‐ms pulses, and 150 ms was the highest pulse duration used. Fifty‐eight percent of papers chose to employ square‐ or rectangular‐shaped pulses and, contrastingly, the remaining papers failed to describe the pulse shape used or if it differed from the commonly used square or rectangular pulse. While not always explicitly described in the reviewed publications, the choice of when and for how long to apply electrical stimulation is vitally important. A third of papers adopted continuous stimulation, whereby stimulation was initiated at or closer to the start of cell culture and was not stopped from that point. A quarter of papers chose intermittent application of their protocols, where stimulation was applied on and off consistently throughout cell culture. Finally, the majority of papers, 42%, employed what we describe as terminal stimulation, in that the protocols were only applied at the end of cell culture in order to trigger observable changes in cells.

### Experimental outcomes

All papers describe, in one way or another, that cells grew preferentially on conductive scaffolds compared to nonconductive controls, though some adverse or undesired effects were seen with increased concentration of the conductive component^34^ or changes to the scaffold architecture.^31^, ^35^ Eight out of 12 papers made specific reference to cell viability, with a variety of tests used and described previously, and all reported control‐comparable or increased viability on conductive scaffolds. Four of the 12 publications also investigated the proliferative state of CMs, though only three of the four described the exact technique used, and found that scaffold conductivity, and in some cases electrical stimulation, improved cell proliferation. One publication only used the amount of cell death or apoptosis to describe the state of CM health on their scaffolds instead, with no mention of viability or proliferation in the manuscript. In this case, random, isotropic nanoscale scaffold topography induced higher cell death when compared to aligned and control scaffolds.^31^ Maturation is highlighted by 8 out of the 12 papers as an important characteristic that scaffolds and electrical stimulation should encourage in engineered CM tissues. Observations and techniques used to assess maturity vary between publications, and in some papers, descriptions of cell maturation are quite ambiguous and appear to only be backed up by microscopic assessment of CMs. Common observations of cell maturity include CM elongation and alignment, and improved or well‐defined organization, alignment, or striation of sarcomeric proteins. A single paper in the review cohort used electrophysiological characteristics to assess CM maturity, with cells on their scaffolds displaying more negative resting potential, increased action potential amplitude, and shorter action potential duration.^31^ IF was used by 11 out of 12 papers and of those, 91% stained for the gap junction protein Cx43. Forty percent of those articles observed an increase in Cx43 expression in CMs cultured on conductive scaffolds, with or without electrical stimulation. Thirty percent described intercellular Cx43 staining along the border between CMs, likely as the result of gap junction formation. Contrastingly, 40% also described Cx43 staining as being homogeneous. Cardiac α‐actinin was another common protein used and was employed by 82% of papers that used IF. Two thirds of researchers described similar results, where staining of CMs on conductive scaffolds revealed well‐defined, organized, striated, or aligned sarcomeric structures. Forty‐four percent of articles described CM organization, shown by α‐actinin staining, as being interconnected, which is likely to mean that sarcomeric structures continue or are connected between individual CMs. Electrical stimulation was frequently used as a tool by researchers to control the contractile behavior of their engineered tissues, with 7 out of 12 papers reporting induced synchronicity of contractions between CMs and control over beating rates as a result of the application of stimulation protocols. However, a minority of articles, 42%, repeated key experimental techniques, excluding simple microscopic observations, before and after stimulation to interrogate the effects on CMs when scaffolds were electrically active. A third of papers described an increase in Cx43 expression after the application of electrical stimulation compared to unstimulated and nonconducting scaffolds. A third of the cohort also reported that the use of electrical stimulation had no significant effect on CM viability, with two publications showing that stimulation increased proliferation of CMs. Additionally, 3 out of 12 papers reported that electrical stimulation helped to orient CMs either in parallel to scaffold architecture or to the direction of the electric field.

## DISCUSSION

As previously stated, the two main goals of TE are either to culture transplantable tissue constructs to repair damaged organ systems or to create *in vitro* disease models that are representative of the human body.^4^, ^5^ Many authors speculated on where their research would best be applied in the future and the former TE goal was a popular suggestion, with half of the reviewed publications mentioning *in vivo* application of their TE constructs for myocardial tissue regeneration or repair.^31^, ^34^, ^36^, ^37^, ^38^, ^39^ Other researchers made more generic predictions, highlighting the importance of their work as a foundation for further cardiac TE research. Shin *et al.* suggested that not only could their results lead to the development of tools for cardiac TE but for neurological biomedical applications too.^40^ A continuation of this research in Wang *et al.* used similar biomaterials to create a manta ray–inspired soft robot, where they suggested their designs could be the basis for the development of more functional and complex autonomous cell‐based robots for a number of different applications.^35^

Our review question and search terms used to probe literature databases were developed to intentionally isolate a manageable, topic‐specific cohort of publications for systematic analysis. But in doing so, this also highlighted the limited published material currently available, even after canvassing all the content in three of the largest literature databases—PubMed, Web of Science, and Scopus. After using inclusion, exclusion, and quality appraisal criteria on the unique items yielded by our literature search, only 12 papers remained. It was previously mentioned that cardiac TE and the development of more complex, dynamic biomaterials for that purpose are novel, emerging fields and this finding reinforces that statement. Despite their rarity, the papers found in this review demonstrated high impact and wide recognition from other authors, with an average number of 134 citations, as reported by the publishing journals. Shin *et al.* received the most citations, with 540 since its publication 8 years ago.^37^ This indicates, perhaps, that while impactful and important to the wider research community, few researchers have the means and expertise to pursue research that spans so many specialist fields. Further to this, the cross‐disciplinary nature of the research can result in nonstandard or inconsistent language, which cause some publications to be missed by search terms when only the title and abstract were canvassed in searching literature databases. Pioneering work by Dvir *et al.*, cited by 8 out of 12 reviewed papers, and work by Tsui *et al.* published immediately before database inception were not captured by our search methodology despite fulfilling exclusion and inclusion criteria.^41^, ^42^

### Effects of electrical stimulation and its different parameters

Seeing as the intention of delivering electrical stimulation in TE is to emulate the same stimulus that CMs receive *in vivo*, it would be prudent to relate parameters like voltage, frequency, pulse shape and duration, and stimulation delivery and duration to the features of cardiac electrophysiology. Beginning with voltage, CMs at rest have a membrane potential of approximately −90 mV and CM action potentials are activated when the membrane potential exceeds −70 mV, known as the threshold potential.^43^ Given this, in theory, any increase in membrane potential greater than 20 mV should initiate CM contraction. However, and though a range of voltages were used, researchers used up to 7 V/cm, which greatly exceeds the ΔV of 20 mV. Having said this, these tissue scaffolds are not perfect conductors, and the conductive components are usually dispersed at reasonably low concentrations–normally the minimum concentration to achieve a conductive percolating network.^44^, ^45^ This acknowledgment, in conjunction with other factors associated with electrical stimulation in cell culture, means that these types of systems have high resistance, in many cases referred to as scaffold impedance, so the voltage used does not equate to the voltage delivered at the cellular level. This was well demonstrated by Shin *et al.* when electrical stimulation was applied parallel and perpendicular to the alignment of CNT forest electrodes imbedded in the gelatin methacrylate (GelMA) scaffold, and in the parallel configuration, a lower voltage was required to elicit CM contractions.^40^ This results in somewhat limited control over the precise voltage delivered to CMs, so instead, ranging from initiating contractions to causing cytotoxic effects, almost any voltage can be applied as long as the scaffold impedance is not high enough to induce negative effects. For neonatal rat CMs, which were used by the majority of publications, it has been recommended that electric field strength does not exceed 8 V/cm.^27^ Multiple publications used a range of electric field strengths in testing electrical stimulation on engineered tissues,^35^, ^37^, ^38^ though some tested this beforehand, setting the protocol voltage to “the minimum voltage required to observe contraction,” as was previously mentioned in the Results.^33^ By varying voltage, researchers were also able to reveal electrophysiological characteristics in different conditions, whereby the excitation threshold of CMs could be compared between scaffolds of different conductivity or when protocol parameters, such as pulse frequency, were changed.^33^, ^37^, ^40^

Pulse frequency is an essential element of electrical stimulation, especially for cardiac TE, as improper frequency can result in cardiac arrhythmia, making engineered tissues unfit for transplantation or making *in vitro* models not comparable to native myocardium. It is fair to say that most researchers envisage their work eventually being utilized in some way in human therapies, though with human CMs being less accessible, the majority of papers resorted to neonatal murine CMs. And while these have been established as an effective analog to human cells, there are still stark differences^46^, ^47^, ^48^—namely that neonatal rat CMs have a resting beating rate around 300 beats per minute (bpm).^49^ The human heart, at rest, can beat anywhere from 60 to 100 bpm. This translates to a significant difference in frequency—1.333 Hz in human versus 5 Hz in neonatal rat CMs. Stimulation protocols should reflect this fact, but in these papers, this is rarely the case, with only 1 of the 11 papers that used neonatal rat CMs actually using a pulse frequency of 5 Hz.^50^ Many publications used a range of frequencies to demonstrate controlled pacing over CM contractions, but the pacing rates used all more closely resembled human rather than murine physiology. While this signifies tissue scaffolds and stimulation protocols developed in these publications would be appropriate for human CMs, they fail to satisfy the requirements of the cell type actually being used. This may have been a limitation of materials and equipment used, but the number of papers that used improper pulse frequencies makes this unlikely. Shin *et al.* reported that CMs were not able to be paced above 2 Hz due to higher voltages required to initiate CM action potentials that the system was not able to accommodate.^38^ Referring back to scaffold impedance, scaffold design and distribution of conductive agents may limit electrical pacing of cells at higher frequencies in many cases, similar to Shin *et al.*

Pulse features are another important part of stimulation protocols in TE, though perhaps considered less in comparison to voltage and frequency. However, proper consideration of pulse application can help to preserve the integrity of the engineered tissues and the systems and scaffolds in and on which they are grown. Duration is a key feature of electrical pulses and differed widely between publications. The majority of publications used 50‐ms pulses, with 2‐ms pulses being second most popular. Mammalian cardiac action potentials can range from 100 to 500 ms; specifically within the ventricles, action potentials generally last 200–300 ms.^51^ This time frame encompasses all stages of the action potential, from rapid depolarization to eventual repolarization and return to resting membrane potential. Typically, the depolarization stage of the action potential, the reaction that electrical pulses are intended to provoke, lasts approximately 2 ms—therefore, pulses need only last that long.^51^ The disadvantages of using prolonged pulses are two‐fold. Sustained current in electrodes for biological systems can result in electrode degradation and charge transfer reactions, where harmful, cytotoxic byproducts are produced,^27^ although this is more true of capacitive coupling stimulation. Additionally, many of the ion channels and pumps that drive cardiac action potentials are voltage dependent, so prolonged increase in voltage may disrupt CM recovery from contraction and decrease their capability of being paced at higher frequencies. Something not often featured in researchers’ descriptions of their protocols is the choice of mono‐ versus biphasic pulses. Much like improper pulse duration, the use of monophasic pulses can induce the production of harmful byproducts and local increases in temperature.^27^, ^52^, ^53^ Monophasic pulses can be likened to DC current, where the flow of electrons only moves in one direction. While biphasic pulses are equivalent to AC current, the flow of electrons in one direction is followed by an equivalent voltage in the opposite direction. Biphasic pulses can, therefore, reverse charge transfer reactions and increase the biocompatibility of electrical stimulation, especially over long courses of time.^52^, ^54^ Those that did describe pulses in this way only stated the use of biphasic pulses.

This review found that the modal average protocol comprises 1–3 V square biphasic 50‐ms pulses at 1 Hz, with stimulation occurring toward the end of cell culture, that is, terminally. While variations of these parameters still yielded positive results, there lacks some consideration for physiological relevance to achieve the optimal protocol, principally in pulse frequency and stimulation application and duration. Based on the conclusions above, we recommend continuous or intermittent stimulation using a protocol of 1 V square biphasic 2‐ms pulses with frequency ranging between 1 and 1.333 Hz for human‐derived CM constructs.

### Effects of conductive scaffold design and materials

While the main aim of this discussion is to review the use of direct coupling electrical stimulation in the collected papers, the conductivity of the scaffolds had significant positive effects on CMs even in the absence of said stimulation. In fact, the focus for many papers in this review was to showcase the novelty of their scaffolds and demonstrating the advantages of their work over standard nonconductive scaffolds, with electrical stimulation more often than not being used to embellish the functionality of these novel tissue scaffolds as an interesting footnote rather than an essential tool used throughout CM culture. Comparable or improved CM viability was reported by the majority of papers in the presence of scaffold conductivity and was largely independent of materials used. The most commonly used technique to observe the differences in CM physiology between conductive and nonconductive scaffolds was IF, namely, on proteins Cx43 and cardiac α‐actinin. Cx43 is a connexin protein that forms gap junction structures in intercalated discs in CM tissues.^22^, ^25^, ^55^, ^56^, ^57^ Gap junctions allow the passage of ions and multiple other metabolites from one cell to another and in cardiac muscle.^56^ They permit action potentials to be propagated across the tissue in unison to ensure synchronized contraction, relating to the function of the heart to efficiently pump blood around the body. As such, proper expression and localization of Cx43 to intercalated discs is a useful indicator that cultured CMs are effectively mimicking their native state. Likewise, cardiac α‐actinin is only expressed in cardiac muscle and forms part of the contractile protein complexes in CMs.^58^ Expression of this protein confirms the CM phenotype but also highlights that CM protein organization, and, much like Cx43 IF, is an important tool for many researchers in confirming the likeness of engineered tissues to native myocardium.^48^, ^59^ In total, 11 out of 12 publications reported increased expression and/or improved organization of one or both of these proteins, giving strong indication that the addition of scaffold conductivity in cardiac TE is a key feature that can help researchers achieve the goal of creating representative engineered tissues. Similarly, many papers also commented on improved CM maturity as a result of conductive scaffolds through frequent observations of the presence of increased spontaneous, rhythmic, and synchronous contractions.^58^, ^60^, ^61^ The improvement of CM contractile behavior, which goes hand‐in‐hand with their electrophysiological function, as a result of the inclusion of conductivity into scaffold design represents a major current gap in the knowledge of this field. Without the presence of external electrical stimulation, this query comes down to the differences and interplay between CMs—ionic conductors—and the conductive components of the scaffold—electrical conductors. As yet, this phenomenon has only received speculative explanations, with one publication suggesting that CNTs may modulate the activity of potassium channels, resulting in higher beating rates.^62^ Two publications from Sun *et al.* showed that multi‐walled CNTs affected the assembly of intercalated discs and increased the expression of related proteins like N‐cadherin and Cx43; however, this only relates to the propagation of action potentials between CMs and not their intrinsic initiation.^63^, ^64^

In considering these TE concepts for *in vivo* applications, it is also important to consider the fate of their components, not only the ability to perform their desired function. As previously stated, there is a clear desire among these publications to design TE scaffolds capable of being used as a therapy for repairing or regenerating damaged myocardium. Clinical application of TE scaffolds comes with serious ethical considerations, principally to do no harm in patients.^65^ A point of weakness in these TE concepts is the fate of either the products from biodegradation or escaped materials from TE formulations. CNTs, gold, and silver are all considered chemically inert but bulk gold and silver are also considered biologically inert;^66^, ^67^ however, these materials take on new properties and behaviors at the nanoscale, and all these materials have been found to possess varying levels of cytotoxicity when used at these size scales.^67^, ^68^, ^69^, ^70^, ^71^, ^72^, ^73^ Of course, these cytotoxic effects can be limited by reducing their concentrations, but this would also make scaffolds less or nonconducting. Low‐density conductivity has been achieved with CNTs fixed in confined colloidal matrices, though there are no published instances of these materials being used in cardiac TE; however, it is conceivable to achieve this same intentional distribution of conductive agents in other systems.^45^, ^74^ CNTs grown using chemical vapor deposition form aligned forests, which, in turn, can form aligned sheets that can be used as tissue scaffolds themselves or make up a conducting layer within other biomaterials, as done by Ren *et al.* ^31^ Alternatively, again with the use of CNTs, conducting agents can be immobilized by certain chemical functionalizations. For example, Peña *et al.* used carboiimide‐lysine crosslinking to covalently bond multi‐walled CNTs to a polymeric reverse‐thermal gel.^75^ Other biomaterial components that have a more structural role in tissue scaffolds are frequently biodegradable, such as poly(ethylene glycol), collagen, and gelatin, which presents its own problems. Once the scaffolds are implanted, over time, only the nonbiodegradable elements would remain, and the scaffold would cease to perform as it was tested *in vitro*. Only one of the papers found by this review appears to have created a scaffold capable of sustaining CMs and delivering electrical stimulation and which is also biodegradable, namely, melanin.^34^ However, from the results of this paper, some of the most concerning instances of cytotoxicity by the conducting component were found.

## CONCLUSION

Electrical stimulation by direct coupling through conductive scaffolds for CM tissue culture may be characterized as a niche scientific topic, as evidenced by the limited number of publications gathered by this systematic review, but this does not diminish its significance to the broader field of cardiac TE. The rarity of applicable publications does, however, make discrete results difficult to compare. Despite researchers often using the same or similar cell types, there were vast differences in scaffold materials and experimental techniques. While the authors of this review strived to make honest comparisons between different research efforts, it must be acknowledged that direct comparisons between publications would be misleading. Nevertheless, progress in this field, and the technologies associated with it, is bringing coherence and consistency to research methodologies from disparate groups and institutions. To that aim, this review has identified the ideal protocol for use in the assessment and stimulation of human cardiac tissue constructs on conductive scaffolds, with the hopes of making results from isolated publications more comparable.

While many published findings from different TE concepts in this review show great promise, much of the research is still in its infancy and will require more time and development to fulfill the outcomes mentioned in this review and deliver an efficacious product or therapy. It is clear that there is strong advantage to developing tissue scaffolds that possess even moderate levels of conductivity. This added feature, when compared to standard tissue culture methodologies, was shown to modulate CM behavior and protein expression. Consistent observations included improved cell viability, increased beating rates, higher expression of Cx43, and improved organization of structural proteins like α‐actinin. With the inclusion of conductivity into tissue scaffold design comes the possibility of stimulating cultures electrically—a vitally important asset in cardiac TE—and even the potential to record electrophysiological activity of CMs.^36^, ^76^ Despite the utility of electrical stimulation for cardiac cell types, researchers frequently only used the technique to embellish results following materials science breakthroughs in the tissue scaffold itself. Equal to the presence of conductivity alone, electrical stimulation proved to be a potent tool to further modulate the characteristics of CM cultures. In addition to enhancing effects seen by scaffold conductivity, stimulation protocols were shown to improve cell proliferation, enable pacing of CM contractions, improve beating rates, and even induce whole cell alignment of CMs in the direction of the electric field—which has significant implications for the assembly of cardiac tissues.^31^, ^34^, ^53^, ^77^ Irrespective of the proposed fate of these TE constructs, the aim was to create a contiguous, functional, organized, and biologically representative tissue layer of CMs. Innovations in biomaterials have contributed the most toward this aim across the reviewed publications and now is the time for researchers to explore, or continue to develop, additional functionalities. Publications have shown the utility of biochemical and biological stimuli, such as growth factors, cytokines, chemokines, and even different cell types;^75^, ^78^ however, it is now time to exploit the capabilities provided by material properties like scaffold architecture, micro‐ and nanoscale topography, and electrical and mechanical stimulation to deliver more complex, dynamic, and innovative cardiac TE technologies.

## COMPETING INTERESTS

The authors declare no competing interests.

## AUTHOR CONTRIBUTIONS

Conceptualization, L.S., K.E., K.J., I.J. and R.L.; methodology, L.S., K.J. and R.L.; formal analysis, L.S.; investigation, L.S.; resources, L.S.; data curation, L.S.; writing—original draft preparation, L.S.; writing—review and editing, L.S., K.E., K.J., I.J. and R.L.; visualization, L.S., K.E., K.J., I.J. and R.L.; supervision, I.J., K.J. and R.L.; project administration, R.L.; funding acquisition, K.J., I.J. and R.L. All authors have read and agreed to the published version of the manuscript.

### PEER REVIEW

The peer review history for this article is available at: https://publons.com/publon/10.1111/nyas.14812.
